# A Study of Practical Proxy Reencryption with a Keyword Search Scheme considering Cloud Storage Structure

**DOI:** 10.1155/2014/615679

**Published:** 2014-02-12

**Authors:** Sun-Ho Lee, Im-Yeong Lee

**Affiliations:** Department of Computer Software Engineering, Soonchunhyang University, Asan-si, Chungcheongnam-do, Republic of Korea

## Abstract

Data outsourcing services have emerged with the increasing use of digital information. They can be used to store data from various devices via networks that are easy to access. Unlike existing removable storage systems, storage outsourcing is available to many users because it has no storage limit and does not require a local storage medium. However, the reliability of storage outsourcing has become an important topic because many users employ it to store large volumes of data. To protect against unethical administrators and attackers, a variety of cryptography systems are used, such as searchable encryption and proxy reencryption. However, existing searchable encryption technology is inconvenient for use in storage outsourcing environments where users upload their data to be shared with others as necessary. In addition, some existing schemes are vulnerable to collusion attacks and have computing cost inefficiencies. In this paper, we analyze existing proxy re-encryption with keyword search.

## 1. Introduction

Network development has accelerated data communication, and data outsourcing services have been developed to store data in distant storage media, which can be retrieved by a user with various devices. Many companies are now providing competitive high-capacity storage services. Thus, an increasing number of people are using storage outsourcing services to store their data. However, the storage of sensitive data such as medical or financial information increases the development of the “Big Brother problem” and the risk of data disclosure by attackers and unethical administrators.

One scheme for protecting user data is data encryption on the data outsourcing server. However, this approach can cause difficulties during data access. Users must download all of their own data, and decryption needs to be applied to the entire dataset before the data can be searched. This can be viewed as a major disadvantage of data outsourcing. Therefore, searchable encryption systems have been developed that can encrypt data indexes to allow index searching without exposing the data to attackers and unethical administrators.

The study of searchable encryption systems began with searchable symmetric encryption (SSE) based on symmetric key cryptography as well as the development of generic cryptographic algorithms. The first construction of SSE was proposed by Song et al. [1]. Then, a new scheme using the Bloom filter schemes was proposed by Goh [2]. In order to provide faster retrieval time, an SSE scheme using an encrypted linked list scheme was announced by Curtmola et al. [3].

Next, research into searchable encryption systems based on a public-key has been actively carried out. The first public key encryption with keyword search (PEKS) using a bilinear map was proposed by Boneh et al. [4]. The PEKS scheme provides a variety of functions; for example, multiuser capability was proposed [5–11].

However, this scheme is difficult to apply in a cloud environment where there is frequent data sharing among users. To address this problem, a proxy reencryption with keyword search (PRES) system has been developed that reencrypts encrypted indexes and allows users to search during safe data storage outsourcing and sharing without the need for a decryption process [12–14].

However, some existing systems do not consider users who share data with other users or the storage outsourcing structure, which means that they handle the indexes and data encryption as a single process. In reality, the indexes and data are stored separately during storage outsourcing. The indexes are stored on the master server, and the data are split into chunks, which are then distributed to many chunk servers. Therefore, searchable reencryption systems are difficult to apply to a real outsourced storage system. In addition, some existing schemes are vulnerable to collusion attack. Some existing schemes allow only one-hop data sharing. In reality, there is no longer any control after the data have been shared. If data need to be shared, the user has no choice other than to accept multihop reencryption. Most searchable reencryption schemes require large volumes of computing resources for data storage and sharing.

The present study examined the operation process of PRES, which operated in the same manner as the above scenario, and analyzed the consequences of a relevant scheme for collusion with an administrator of an untrusted remote storage and sharing target.

## 2. Preliminaries

In this section, we provide the necessary preliminary details.

### 2.1. Bilinear Maps

The bilinear map was proposed originally as a tool for attacking elliptical curve encryption by reducing the problem of discrete algebra on an elliptical curve to the problem of discrete algebra in a finite field, thereby reducing its complexity. However, this scheme has been used recently as an encryption tool for information protection, instead of an attacking tool. Bilinear pairing is equivalent to a bilinear map. These terms are defined and the theory is described below.

**Definition 1**

Characteristics that satisfy an admissible bilinear map are as follows.

- Bilinear: define a map *e* = *G* × *G* → *G*_*T*_ as bilinear if *e*(*P* ^*a*^, *P* ^*b*^) = *e*(*P*,*Q*)^*ab*^ where all *P*, *Q* ∈ *G*, and all *a*, *b* ∈ *Z*.
- Nondegenerate: the map does not relate all pairs in *G* × *G* to the identity in *G*_*T*_. Note that *G* and *G*_*T*_ are groups of prime order, which implies that if *P* is a generator of *G*, *e*(*P*, *P*) is a generator of *G*_*T*_.
- Computable: there is an efficient algorithm to compute *e*(*P*, *Q*) for any *P*, *Q* ∈ *G*. The following definition was constructed based on the bilinear map *e*(*P* ^*a*^, *Q* ^*b*^) = *e*(*P*,*Q* ^*b*^)^*a*^ = *e*(*P* ^*a*^,*Q*)^*b*^ = *e*(*P*,*Q*)^*ab*^ = *e*(*P* ^*ab*^, *Q*) = *e*(*P*, *Q* ^*ab*^). With this map, the D-H decision problem can be solved readily for ellipses using the following equation: *e*(*P* ^*a*^, *Q* ^*b*^) = *e*(*P* ^*c*^, *P*)⇒*ab* = *c*. Therefore, the following is the basis for resolving the difficulties of the bilinear map, which is used as an encryption tool by many encryption protocols.

**Definition 2**

When the elements *G*, *P*, *P* ^*a*^, *P* ^*b*^, *P* ^*c*^ (BDHP, Bilinear Diffie-Hellman Problem) are given, this relates to the *e*(*P*,*P*)^*abc*^ calculation problem. In this study, the admissible bilinear map was used as the basis for secret number production during the key construction process between heterogeneous devices. This problem can be solved if the ellipse curve discrete mathematics problem can be solved. For example, a can be calculated from *P* ^*a*^, so *e*(*P*,*P*)^*abc*^ can be calculated using *e*(*P* ^*b*^,*P* ^*c*^)^*a*^.

### 2.2. Existing PRES Scheme

Let us take a look at [13] proposed by Chen and Li in 2011.

#### 2.2.1. Notation

The notation used in this scheme are as follows.

- *q*: Prime number.
- *G*_1_: Cyclic additive group of order *p*.
- *G*_2_: Cyclic multiplicative group of order *p*.
- *g*: Generator of *G*.
- *e*: Bilinear map, *G*_1_ × *G*_1_ → *G*_2_.
- *H*_1_( ): Hash function, {0,1}* → *G*_1_*.
- *H*_2_( ): Hash function, *G*_2_ → {0,1}^log⁡*q*^.
- *H*_3_( ): Hash function, {0,1}* → *G*_1_*.
- *H*_4_( ): Hash function, *G*_2_ → {0,1}^*n*^.

#### 2.2.2. Protocol

As with most PRES schemes, the protocol of Chen et al. had a total of 7 phases: KGen, Enc, RKGen, REnc, TGen, Test, and Dec.

*KGen Phase*. Objects each public/private open key pairs using remote storage in the KGen stage:

(1) Alice:  Apub=ga,  Apriv=a∈ZpBob:  Bpub=gb,  Bpriv=b∈ZpServer:  Spub=gs,  Spriv=s∈Zp.

*Enc Phase*. User *A* transmits encrypted data to remote storage *S*:

(2) r∈Zq∗ρ∈{0,1}nu1=hrAlice:   u2=ρ⊕H4(e(ha,gs)r)u3=m·e(H3(ρ),ga)rCWi=H2(e(ga,H1(Wi))r)Cm=(u1,u2,u3)  Alice⟶Server:  CW1,CW2,…,CWk,Cm.

*RKGen Phase*. User *A* transmits a reencryption key to *S* in order to share data with *B*:

(3) $$\begin{array}{c} \text{Alice}⟶\text{Server:}\;\;rk_{A\to B}=g^{abr}. \end{array}$$

*REnc Phase*. *S* reencrypts the data with the reencryption key transmitted by *A*.

(4) ρ=u2⊕H4(e(ha,gs)r)  =u2⊕H4(e(hr,ga)s)Server:u4=e(H3(ρ),rkA→B)  =e(H3(ρ),gabr) CB=(u3,u4).         

*TGen Phase*. User *B* transmits a produced trapdoor to *S* in order to search the shared data from *A*:

(5) $$\begin{array}{c} \text{Alice}⟶\text{Server:}\;\;T_{W_{j}}=H_{1}{\left(W_{j}\right)}^{1/b}. \end{array}$$

*Test Phase*. *S* transmits the search results after searching the data using the trapdoor sent from *B*.

(6) Server:  CWi=?H2(e(rkA→B,TWj))=H2(e(gabr,H1(Wj)1/b))=H2(e(ga,H1(Wj)r)).

*Dec Phase*. User *B* verifies the contents by decrypting the data relevant to the search results:

(7) Bob:  u3(u4)1/b=m·e(H3(ρ),ga)r(e(H3(ρ),gab)r)1/b=m·e(H3(ρ),ga)re(H3(ρ),ga)r=m.

#### 2.2.3. An Analysis on the Protocol

We will analyze the PRES scheme proposed by Chen et al. for possible security threats.

*Analysis 1: Problem of Sharing Process.* In RKGen phase, *A* produced *rk*_*A*→*B*_ = *g* ^*abr*^ to share his own data. This is known as producing with the similar scheme of (*g* ^*b*^)^*ar*^. However, the value of *r* is not knowable even if the data owner is *A*. In the Enc phase, *A* produces a random *r* value according to different data and does not save it separately. In addition, directly deducting the *r* value used only in a multiplication operation from the encrypted data is not possible even if *A* is the data owner. In other words, *A* cannot produce an *rk*_*A*→*B*_ = *g* ^*abr*^ value to reencrypt the uploaded data. In order for RKGen to be established, the *r* value should be opened, or *A* should save all *r* values relevant to each data set.

*Analysis 2: Collusion Problem.* Let us suppose that *S* and *B* are in collusion. If the *r* value is revealed, or all files are encrypted using the same *r* values, *S* can easily produce the reencryption key *rk*_*A*→*B*_′ = (*g* ^*a*^)^*br*^ by using the open key of data owner *A* and the personal key of colluder *B*. Then, a file that is not a sharing object can be reencrypted as below for *B*:

(8) ρ=u2⊕H4(e(ha,gs)r)  =u2⊕H4(e(hr,ga)s)Server:u4=e(H3(ρ),rkA→B′)  =e(H3(ρ),gabr) CB=(u3,u4).         

According to the above scheme, unwanted sharing not just with *B* but with anybody that *A* did not want is possible.

*Analysis 3: Data Encryption Problem.* In Enc phase the process *u*_3_ = *m* · *e*(*H*_3_(*ρ*),*g* ^*a*^)^*r*^ is implemented to encrypt the data. In other words, a multiplicative group encrypts messages by multiplying the element and the message in an elliptic curve situation. The multiplication operation of the elliptic curve is only possible with elements of the multiplicative group. In other words, when changing a multiplicative group of plaintext to an element, obtaining a normal plaintext value is not possible during decryption in the future.

### 2.3. Security Requirement

The following requirements should be met to ensure safe searching and sharing in an outsourced storage environment.

**Figure 1:**
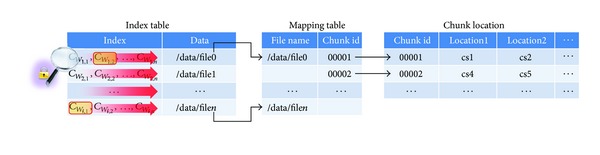
Existing index structure of PRES.

**Figure 2:**
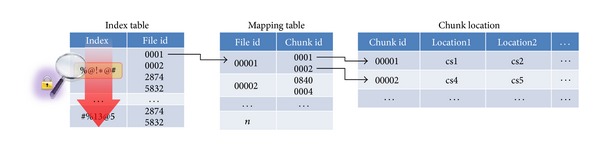
Proposed index structure of PRES.

- Confidentiality: data transmitted between the outsourced storage server and client terminal should be identifiable only by validated users.
- Search speed: a client who has limited system resources should be able to search documents quickly, including word processing files, stored in outsourced storage systems. In the case where the data index structure of the existing scheme is the same as in Figure 1, and the server needs to retrieve data from all indexes to find the data containing the keyword, it is very inefficient. In addition, many previously developed search algorithms do not apply to this structure, so the storage server must perform a sequential search. In this structure, the scan speed decreases rapidly with an increasing number of documents. In order to solve this problem, the structure of the encryption index must be changed, as shown in Figure 2. If we adopt such a structure, the previously developed fastest search algorithm can be used for the data search.
- Traffic efficiency: communication volume between the client and server should be small for energy and network resource efficiency.
- Calculation efficiency: calculation efficiency should be provided for index generation, search execution, and safe sharing of data with other users. The previous scheme is highly inefficient for encrypting variable-length data. Data encryption is performed with a symmetric key in a multiplicative group, and hiding the encrypted key using a multiplying operation is more effective.
- Storage volume efficiency: a variety of distributed file systems have been developed to provide cloud storage services. These systems store the index in master server's memory for faster data retrieving. In other words, the storage capacity of the index has limitations. Due to these circumstances, a service provider uses this technique to merge the repeated keyword and optimize the index. The server cannot merge duplicated keywords, in the case of existing schemes, using the same structure as in Figure 1. In this the structure the index capacity will also increase rapidly depending on the number of documents. However, if we adopt the structure shown in Figure 2, index capacity management will be more efficient.
- Sharing efficiency among users: encrypted data must be retrieved from saved remote data and be securely and efficiently shared with those users who use an unreliable server. Cloud service providers should make shareable only the data that the data owner wishes to share with another user. The PRES papers most often propose previously used proxy reencryption (PRE). These schemes provide a once-only sharing function. In other words, *B* cannot share data with another user *C* with a similar scheme as the one used to share the data between users *A* and *B*. However, *B* is able to search and decrypt the shared data and then share it by saving it to the remote storage again through the PRES encryption process. The existing PRES is not sharing the shared data to *B* again, and additional decryption and encryption operations are needed to share the data again. Therefore, PRES needs to consider a re-share operation.
- Prevention of a collusion attack: the administrator of the remote storage is treated as an untrusted object, and the administrator may obtain unauthorized access to the data through collusion. Therefore, PRES proposed in the future needs to be safe from collusion attack.

## 3. Proposed Scheme

**Figure 3:**
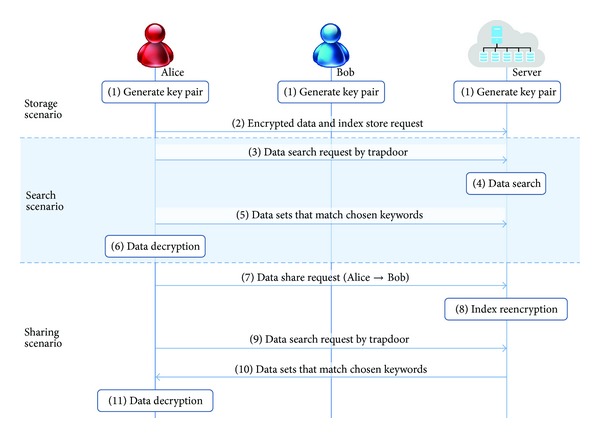
Flow chart of proposed scheme.

In this paper, a practical proxy reencryption scheme with a keyword search capability is proposed considering the structural characteristics of an entrusted cloud storage center. This paper describes what steps should be taken in a secure data storage, searching, and sharing scenario (refer Figure 3).

### 3.1. Notation

- ||: Concatenation.
- *p*: Prime number.
- *n*: Number of data.
- *m*: Number of keyword on data.
- *G*: Cyclic additive group of order *p*.
- *G*_*T*_: Cyclic multiplicative group of order *p*.
- *g*: Generator of *G*.
- *e*: Bilinear map, *G* × *G* → *G*_*T*_.
- sk_∗_: ∗'s private key in *Z*_*p*_.
- pk_∗_: ∗'s public key in *G*.
- pd_*i*_: *i*th plain data.
- ed_*i*_: *i*th encrypted data.
- *k*_*i*_: *i*th data encryption key (*i* = 1 ~ *n*).
- *w*_*i*,*j*_: *j*th keyword on *i*th data (*j* = 1 ~ *m*).
- Enc_*k*_( ): Symmetric key encryption by key *k*.
- Dec_*k*_( ): Symmetric key decryption by key *k*.
- *W*_*i*_: Set of keyword on *i*th data ∗.
- *H*_1_( ): Hash function, {0,1}* → *G*.
- *H*_2_( ): Hash function, *G* → *G*.
- *T*_∗_: Trapdoor searching keyword ∗.

### 3.2. Definition

The detailed steps performed by the proposed scheme are as follows.

- KeyGen: the users of the outsourced storage generate public key pairs prior to using the service. The storage outsourcing server should not store the user's private key. If the private key is leaked, an attacker can generate a trapdoor by acting as the owner of the private key. Thus, we generate a key pair based on the discrete logarithm problem (DLP).
- Enc(sk, *W*, pd) → *E*, ed: the data owner creates the encrypted index, *E*, and encrypted data, ed, which only the owner can search by inputting his or her own private key, sk, and a set of keywords, *W*, which are sent to the master server.
- TGen(sk, *w*) → *T*_*w*_: to search the data safely, the user creates a trapdoor, *T*_*w*_, which does not leak information related to the keyword *w*, which is being searched for using the private key sk. The trapdoor is sent to the master server. The storage outsourcing administrator should not be able to access information via a trapdoor.
- Test(*E*, *T*_*w*_)→“yes” or “no”: using the trapdoor generated by the user's private key and the search keyword, the server performs a test to confirm whether the encrypted data contain the keywords. If the cipher text contains the keyword specified, the server sends a “yes” to the user and a “no” if it does not. Thus, the server cannot learn anything about the keywords or the data.
- RKGen(sk_*a*_, *h*(sk_*b*_)) → *rk*_*a*→*b*_: the data owner *A* creates a reencryption key, *rk*_*a*→*b*_, to create a data index for sharing that *B* can search. The reencryption key is created with the data owner's secret key sk_*a*_, and the hashed secret key *h*(sk_*b*_) of the user who will be sharing the data.
- REnc(sk_*a*_, pk_*b*_, *E*_*a*_) → *E*_*b*_: the data owner *a* creates a parameter to generate a data index for sharing that can be searched by *b*. This parameter is created using the data owner's private key sk_*a*_ and the public key pk_*b*_ of the user who will be sharing the data. The master server creates a new index, *E*_*b*_, which *b* can use to search via the trapdoor.
- Dec(sk, *E*, ed) → pd: the rightful owner of the encrypted data uses their private key to decrypt the encrypted data.

### 3.3. Storage Scenario

**Figure 4:**
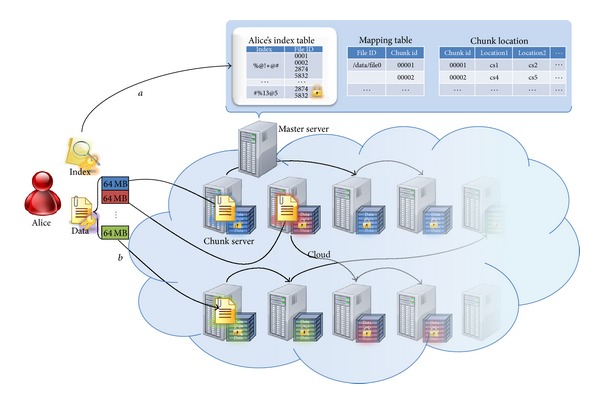
Storage scenario.

The proposed scheme considers the outsourced storage structure so an encrypting index used for sharing and searching is stored on the master server. We assume that each user has received a key pair before using the storage outsourcing service (refer to Step 1). The user encrypts the necessary keywords during data searching so they can perform their own search later and send this to the master server (refer to Step 2). The master server sends chunk information to the user for data storage, who then divides the data into chunks and stores it on the designated chunk server (see Figure 4).

**Step 1 (key generation (KeyGen))**

Each storage outsourcing service user generates a key pair:

 
*x* ∈ *Z*_*p*_ selection
 sk = *x* setting up
 pk = *g* ^*x*^ setting up.

**Step 2 (index and data encryption (Enc))**

The data owner generates an encrypted index which can be used for searching securely:

 Alice: *k*_*i*_ selection
 
*ap* = pk_*a*_^*h*(*k*_*i*_)^
 
*ew*_*i*,*j*_ = *e*(pk_*s*_,*H*_1_(*w*_*i*·*j*_))^*h*_2_(*h*_2_(sk_*a*_)||*w*_*i*,*j*_)^
 
*EW* = {*ew*_*i*,1_, *ew*_*i*,2_,…, *ew*_*i*,*m*_}
 
*EK*_*i*_ = *e*(*H*_2_(pk_*s*_),*g*)^*hk*^ · *k*_*i*_ output encrypted index for the master server
 
*ED*_*i*_ = Enc_*k*_*i*__(*PD*_*i*_) output encrypted data for the chunk server
 
*A* → *S*: *EW*, *EK*_*i*_, *ED*_*i*_.

### 3.4. Search Scenario

**Figure 5:**
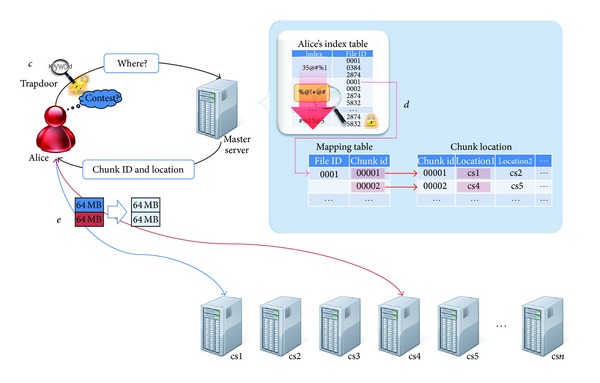
Search scenario.

The user sends a trapdoor that can search data without exposing keyword information to the master server (refer to Step 1). The master server searches for the data with the keyword in the encrypted index using the trapdoor and then sends the chunk information that corresponds to the data to the user (refer to Step 2). The retrieved data is decrypted by the legitimate user (refer to Step 3). The user acquires the data by summing each chunk received from the chunk server that stores the data (see Figure 5).

**Step 1 (trapdoor generation (TGen))**

A user, *a*, who wants to search the data generates a trapdoor using the keywords and his or her secret key:

 Alice → Server: *T*_*w*_ = *H*_1_(*w*)^−sk_*a*_^||*h*_2_(*h*_2_(sk_*a*_)||*w*).

**Step 2 (Test)**

To confirm that the data contains the keywords sought by the user, the user performs the following tests with the public key, trapdoor, and crypt obtained from the server:

(9) Server:    ew=?e(pkas,H1(w)−ska)h2(h2(ska)||w)  =e(ga·s,H1(w)−a)h2(h2(ska)||w)=e(gs,H1(w))h2(h2(ska)||w)=e(pks,H1(w))h2(h2(ska)||w)

**Step 3 (decryption (Dec))**

The user can perform the following decryption using their private key and the crypt obtained from the server:

(10) Alice:  ki=EK/e(ap,H2(pks))−ska=EK/e(pkah(k),H2(pks))−a=EK/e(gh(k),H2(pks)):  output  decryption  keyPDi=Decki(EDi):  output  decrypted  data.

### 3.5. Sharing Scenario

**Figure 6:**
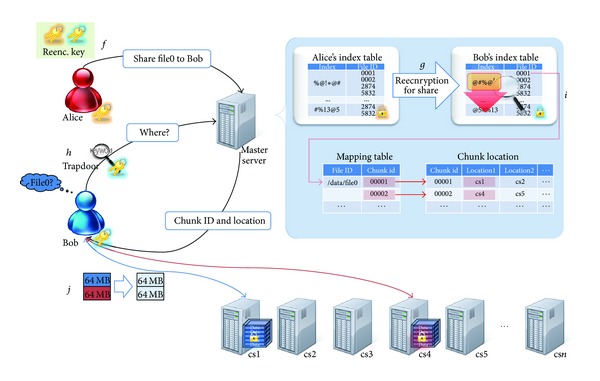
Sharing scenario.

To share data with the desired user and to allow the shared users to share data freely with another user, reencryption needs to be performed to allow the shared users to search only the encrypted index. Many parameters are required to implement proxy reencryption and a separate searchable encryption scheme for secure data sharing in a storage outsourcing environment, which reduces the storage volume efficiency. Therefore, we propose an algorithm that provides both functions simultaneously. First, parameter *A* is generated to allow index sharing with another user, which is sent to the storage outsourcing provider by the owner of the data (refer to Step 1). Next, the storage outsourcing provider changes the owner's index with respect to the data sharing target. Shared (reencrypted) data searching is then possible, as shown in Steps 2–5. A user who acquires the data sharing index can always search for the corresponding data using keywords and then download it (see Figure 6).

**Step 1 (reencryption key generation (RKGen))**

If the data owner wants to share data with other users, he or she can generate keys for reencryption. If user *a* wants to share data with user *b*, *a* generates parameter *A*′ using *a*'s secret key and *b*'s public key, as follows:

 Bob → Alice: *h*(*b*)
 Alice: *rk*_*a*→*b*_ = *h*_2_(*h*_2_(*b*), *w*_*i*·*j*_)/*h*_2_(*h*_2_(*a*), *w*_*i*·*j*_)
 
*ap*′ = pk_*b*_^*h*(*k*)^
 Alice → Server: *rk*_*a*→*b*_||*ap*′.

**Step 2 (reencryption (REnc))**

If user *a* wants to share data with user *b*, *a* generates parameter *ew*_*i*,*j*_′ using *a*'s secret key and *b*'s hashed secret key, as follows:

(11) Server:   ewi,jrka→b=ewi,jh2(h2(skb),wi·j)/h2(h2(ska),wi·j)=e(pks,H1(wi·j))h2(h2(skb)||wi,j).

**Step 3 (trapdoor generation (TGen))**

User *b* who wants to search the data, generates a trapdoor using the keywords and his or her secret key:

(12) $$\begin{array}{c} \text{Bob:}\;\;T_{w}=H_{1}{\left(w\right)}^{-{\text{sk}}_{b}}||h_{2}\left(h_{2}\left({\text{sk}}_{b}\right)||w\right). \end{array}$$

**Step 4 (test)**

To confirm that the data contains the keywords the user seeks, the server performs the following tests using bob's trapdoor. It checks the equality *ew* = ?*e*(pk_*b*_^*s*^,*H*_1_(*w*)^−sk_*b*_^)^*h*_2_(*h*_2_(sk_*b*_)||*w*)^. If this is true, the output is “Yes” but “No” if not,

(13) Server:  ew=?e(pkbs,H1(w)−skb)h2(h2(skb)||w)=e(gb·s,H1(w)−b)h2(h2(skb)||w)=e(gs,H1(w))h2(h2(skb)||w)=e(pks,H1(w))h2(h2(skb)||w).

**Step 5 (decryption (Dec))**

The user can perform the following decryption with his or her private key:

(14) Bob:  ki=EK/e(ap′,H2(pks))−skb=EK/e(pkbh(k),H2(pks))−a=EK/e(gh(k),H2(pks)):  output  decryption  keyPDi=Decki(EDi):  output  decrypted  data.    

## 4. Analysis

The proposed scheme satisfies the following requirements.

**Figure 7:**
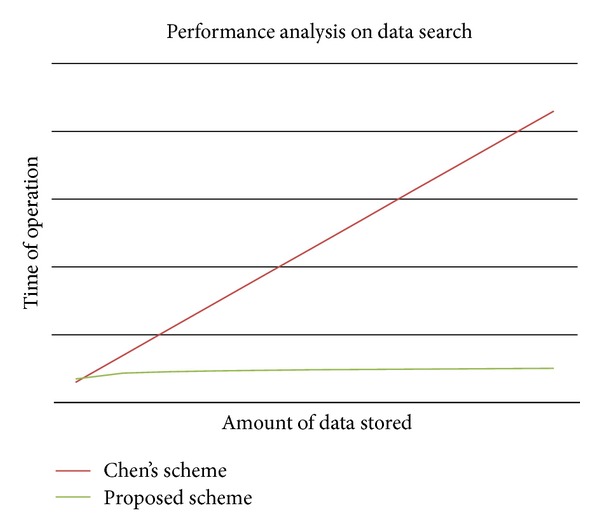
Search speed.

**Figure 8:**
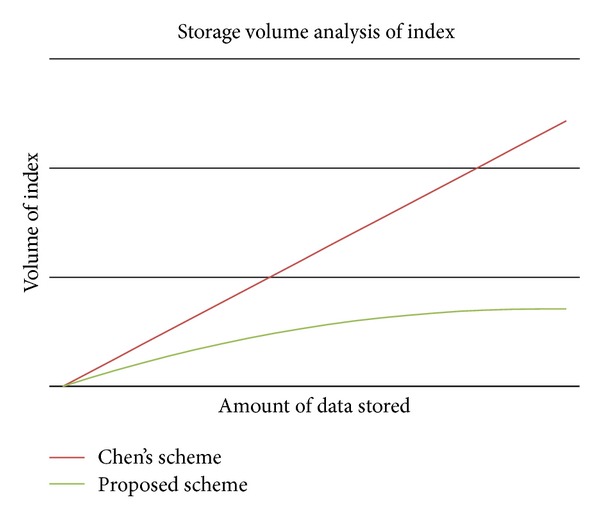
Storage volume.

- Confidentiality: using pairing, the proposed scheme makes it difficult for a malicious third party to decrypt communication contents, even if they eavesdrop on communications between the client and the server.
- Search speed: a quick index search is possible by using the index structure shown in Figure 2, and a user can check whether a document contains keywords by performing single pairing calculations, which increases the searching speed (refer Figure 7).
- Traffic efficiency: keyword search and reencryption requires only one round of communication, so the scheme increases the communication volume efficiency.
- Storage volume efficiency: to use a new index structure, the proposed scheme can reduce storage volume dramatically despite increasing the index document storage space compared to traditional schemes (refer Figure 8). Because, the proposed scheme can merge the same keywords.
- Calculation efficiency: the relatively simple pairing calculation implies that the proposed scheme allows users to generate indexes and search documents, as well as perform reencryption, which increases the calculation efficiency (refer Table 1).
- Sharing efficiency among users: our scheme allows encrypted and stored data on an unreliable remote outsourced storage server to be shared safely and efficiently. In addition, our proposed scheme is different from existing schemes because it does not require the shared subjects to be specified in advance, and no additional devices are required to manage the subjects who receive the shared data. Finally, if users want to re-share the data shared by the owner with other users, they only require one pairing calculation in an unreliable storage outsourcing environment.
- Prevention of collusion attack: in the proposed scheme, each data set is encrypted by a different random key (for symmetric encryption). Therefore the sharing phase can be operated by only the lawful data owner. An unethical administrator cannot use a collusion attack, because the key is known only to the lawful data owner.

## 5. Conclusion

The advent of storage outsourcing services has allowed many users to store and access data. Recent studies of the application of searchable encryption technologies to storage outsourcing have attempted to ensure the security of data. However, most available searchable encryption technologies are inefficient when adding data sharing objects because they are based on e-mail environments, which determine the objects with which data can be shared. In a storage outsourcing environment, users upload data on their own and share the data in a safe manner. Therefore, the indexes and data are separated so available technologies are compatible with data storage outsourcing systems. After considering the requirements of the data storage outsourcing environment, we specified the security requirements and proposed a scheme that provides both functions simultaneously: a proxy reencryption function and a searchable encryption function. The proposed scheme provides a free sharing feature which has the more calculation efficiency than existing schemes. And we adopted the new index structure for fast searching data on cloud storage. It appears that search schemes based on multiple keywords will become important for ensuring flexibility and for facilitating searches during data storage outsourcing. In the future, it will be necessary to develop a reencryption system where an index containing multiple keywords of variable lengths can be encrypted and searched flexibly.

## Figures and Tables

**Table 1:** Calculation efficiency analysis.

	Chen's scheme					Proposed scheme				
	Exponential operation	Pairing operation	Hash operation	Multiply operation	Comparison operation	Exponential operation	Pairing operation	Hash operation	Multiply operation	Comparison operation
Kgen	*u*					*u*				
Enc	2*m* + 4	*m* + 2	2*m* + 2	1		*m* + 1	*m* + 1	3*m* + 1	1	
RKGen	2					2				
ReEnc	2	2	2				1			
TGen	1		1			1		3		
Test		1	1		*n* ∗ *m*	1	1			*c*
Dec	1			1		1	1		1	

*c*: number of comparison operation on existing search scheme (*c* ≤ log⁡_2_(*n*∗*m*)), *m*: number of keyword on document, *n*: number of all documents on cloud storage, *u*: number of user.
